# Positive *H. pylori* status predicts better prognosis of non-cardiac gastric cancer patients: results from cohort study and meta-analysis

**DOI:** 10.1186/s12885-022-09222-y

**Published:** 2022-02-08

**Authors:** Zhifang Jia, Min Zheng, Jing Jiang, Donghui Cao, Yanhua Wu, Yuzheng Zhang, Yingli Fu, Xueyuan Cao

**Affiliations:** 1 Division of Clinical Research, The First Hospital of Jilin University, Changchun, China; 2 Department of Epidemiology and Biostatistics, School of Public Health, Jilin University, Changchun, China; 3 Department of Gastrointestinal Surgery, The First Hospital of Jilin University, Changchun, China

**Keywords:** Gastric cancer, *H. pylori*, Meta-analysis, Prognostic study, Prognosis

## Abstract

**Background:**

Previous researches have associated *Helicobacter pylori* (*H. pylori*) with a prognosis of gastric cancer (GC), however, without a concert conclusion. This study aimed to study this issue further by a prospective cohort study and a meta-analysis.

**Methods:**

Histologically diagnosed gastric cancer (GC) patients were recruited into the primary prospective cohort study between January 2009 to December 2013. All the patients were followed-up periodically to record information on post-surgery therapy and overall survival status. The pre-surgery status of *H. pylori* was measured by enzyme-linked immunosorbent assay. A meta-analysis was conducted after retrieving related researches in the databases of PubMed and Embase up to April 2020. Pooled hazard ratios (HRs) and 95% confidence intervals (CIs) were summarized to validate the relationship between *H. pylori* infection and the survival time of GC patients. *I*^*2*^ statistics and Q test were used to assess the heterogeneity. Sensitivity analyses were performed using Galbraith’s plot, leave-one-out analysis, subgroup analyses and meta-regression to explore the sources of heterogeneity and the stability of the summary results.

**Results:**

A total of 743 GC patients with radical tumorectomy were included prospectively and 516 (69.4%) were positive on *H. pylori*. *H. pylori*-positive patients tended to survive longer than -negative ones (HR 0.92, 95%CI: 0.74–1.15), though the tendency was not statistically significant. Cohort studies on the prognosis of GC were retrieved comprehensively by assessing the full-text and 59 published studies, together with the result of our study, were included in the further meta-analysis. The summarized results related the positive status of *H. pylori* to better overall survival (HR 0.81, 95%CI: 0.72–0.90) and disease-free survival (HR 0.83, 95%CI: 0.67–0.99). Results from subgroup analyses indicated that the pooled magnitude of this association was relatively lower in studies not referring to *H. pylori* in title and abstract.

**Conclusions:**

In conclusion, gastric cancer patients with *H. pylori* have a better prognosis than patients of *H. pylori* negative. More stringent surveillance strategies may be necessary for patients with *H. pylori* negative at cancer diagnosis.

**Supplementary Information:**

The online version contains supplementary material available at 10.1186/s12885-022-09222-y.

## Background

Gastric cancer (GC) is one of the most prevailing cancers and the top three cancer-related death causes worldwide. In 2018, it was estimated that over 782, 000 patients died of gastric cancer, and more than half of the deaths occurred in Eastern Asia [1]. Despite the progress at diagnosis and therapies in recent years, the prognosis of GC is still limited. More studies were warranted to recognize patients at risk of recurrence or death from GC by exploring more biomarkers.

*Helicobacter pylori* (*H. pylori*), a bacterium colonizing in the stomach, was graded as a Group I carcinogen in 1994 by the International Agency for Research on Cancer [2]. Extensive studies have concluded that *H. pylori* infection contributed to gastric cancer, and it is estimated that nearly two-thirds of new gastric cancer cases are attributable to chronic *H. pylori* infection [3] and the eradication of *H. pylori* could reduce the risk of GC [4].

Recently, epidemiological studies have suggested that *H. pylori* infection was also related to the prognosis of GC [5–10]. Some studies have indicated that patients positive for *H. pylori* have better overall survival (OS) compared to patients of negative [5–7]. Other studies reported that *H. pylori* status was not associated with the survival of GC [8–10]. There are also studies showing that *H. pylori* infection has an association with worse overall survival [11–13]. Therefore, the relationship between *H. pylori* infection and the prognosis with respect to GC is still unclear. Previous meta-analyses have examined this issue [14–16]. The most recent study by Fang et al. retrieved the relevant studies that were published before March 2017 and showed that *H. pylori*-positive status was related to longer OS [16]. Recently, more studies on GC prognosis report the status of *H. pylori* infection, with a better understanding of *H. pylori* and the availability of detection methods. However, many prognostic studies did not use the words *H. pylori* in the title, abstract, or keywords sections of the paper [17–19], thus limiting the ability to retrieve these studies in a literature search. This phenomenon is more prominent in studies showing *H. pylori* status is not associated with GC prognosis. Therefore, many studies may be missed when the term *H. pylori* is used as part of the search strategy, which was done by all the previous meta-analyses. Moreover, 3 years have passed since the last meta-analysis, and ever since, dozens of studies on this issue have been published. Therefore, to comprehensively search for the relevant studies and summarize the relationship between the *H. pylori* status and GC survival, we performed this study, in which we combined the results of our primary prospective cohort study with the results from a meta-analysis.

## Methods

### Prospective cohort study

#### Subjects

Gastric cancer patients were recruited from the First Hospital of Jilin University from January 2009 to December 2013. These patients were histologically diagnosed with gastric cancer and hospitalized for curative-intent tumorectomy, without any chemotherapy before surgery. Their demographical and clinicopathological data were collected. Pathological parameters were determined based on postoperative pathologic examination. The histological type was assessed by the criteria of the World Health Organization and categorized as tubular adenocarcinoma, signet ring cell, and others. Histological grade was defined as well-to-moderate differentiated and poorly differentiated. Clinical stages were determined according to the American Joint Committee on Cancer (7th edition) [20]. All the participants signed the informed consent before entering the study.

Patients were prospectively followed-up periodically after being discharged from the hospital. The follow-ups were scheduled for 3 months, 6 months, 1 year after surgery, and annually afterwards. And information on post-surgery chemotherapy, survival status, including death date and death reason if the patients died, were collected. Survival time was defined as the duration from the date of tumorectomy to the date of death. And when the patients were alive or lost to follow-up, the calculation of the period of survival was based on the date of tumorectomy and the date of the latest successful contact. Patients who died within 1 month of surgery were excluded from further survival analysis.

#### Detection of *H. pylori* status

Presurgery levels of serum immunoglobulin G (IgG) antibodies to *H. pylori* were evaluated by enzyme-linked immunosorbent assay (Biohit, Helsinki, Finland). Titers > 30 EIU were counted as positive for *H. pylori* according to the kit instructions.

#### Statistical analysis

Continuous variables like age were described as median with interquartile range and compared by Mann-Whitney U test between *H. pylori-*positive and -negative groups. Categorical variables were presented as frequencies with proportions and compared with Pearson χ^2^ test or Fisher’s exact test. The survival curves were plotted using Kaplan-Meier method and compared by the log-rank test between patients of *H. pylori-positive* and -negative. Multivariate Cox’s proportional hazard regression was utilized to calculate the independent predictive value of *H. pylori* on GC overall survival after adjusting for other potential prognostic factors by estimating the hazard ratios (HRs) with their 95% confidence intervals (CIs). All analyses were performed using SAS software (version 9.4, SAS Institute, NC, USA). A two-tailed *P*-value less than 0.05 was considered statistically significant.

### Meta-analysis

#### Search strategy

A comprehensive search was performed in PubMed and Embase databases for studies concerning patients’ prognosis on gastric cancer published in English with the strategy of (1) “stomach” or “gastric”; and (2) “cancer” or “neoplasm” or “carcinoma” and (3) “survival” or “prognosis”. Then, the full-texts of the retrieved articles were assessed to screen all the potential studies on *H. pylori* and GC prognosis. The search was last conducted on April 10, 2020. Detailed retrieving strategies were attached in the supplementary file 1.

#### Eligibility criteria of the studies

Studies were eligible if: (1) they evaluated the association of *H. pylori* status at diagnosis with a prognosis of gastric cancer; (2) the prognosis was about overall survival (the primary outcome of our interest) or recurrence-free survival (RFS) and disease-free survival (DFS) (the secondary outcomes of interest); (3) the studies designed as cohort studies, irrespective of prospective or retrospective cohort; (4) they reported the HRs and 95% CIs to quantify the association, or there was sufficient information to estimate the relevant HRs and 95% CIs. Studies were excluded if they had overlapping subjects with studies already included.

#### Quality assessment

The quality of each eligible study was evaluated with the modified Newcastle-Ottawa Scale (sTable 1) based on three aspects: selection of subject, comparability between groups, and determination of the outcome. The maximum score was 9, and studies with scores greater than 7 were designated as high-quality. The evaluation was independently performed by two investigators (ZM and ZFJ), and disagreements were resolved by re-evaluating and then discussing with the third investigator (JJ).

#### Data extraction

The following data was extracted from each eligible study: the first author, publication year, geographical location of subjects, demographic characteristics of subjects, clinical stage of the patients, the method to determine *H. pylori*, number of patients positive and negative for *H. pylori*, surgery treatment, duration of follow-up and prognostic evaluation of univariate or multivariate analysis. Two investigators independently extracted all the data (ZM and ZFJ) and disagreements were resolved by group discussion.

#### Statistical analysis

HRs and their 95% CIs were extracted to quantify the prognostic value of *H. pylori* infection on gastric cancer. HRs from multivariate Cox regression analysis were preferred. If unavailable, HRs of a univariate analysis would be extracted. If no HRs were available, HRs with 95% CIs would be estimated using the tool developed by Tierney et al. [21] or estimated from Kaplan-Meier survival curves using Engauge Digitizer(version 4.1, http://digitizer.sourceforge.net). Then HRs were pooled using the random-effect model allowing for heterogeneity among studies. Heterogeneity was evaluated by *I*^*2*^ and the *P-*value of the Q test. If *I*^*2*^ is over 50%, the heterogeneity is thought to be high. If *I*^*2*^ is below 25%, the heterogeneity is thought to be low. Otherwise, the heterogeneity is thought to be moderate. Funnel plots and Egger’s test were applied to examine the potential publication bias of the studies included. Sensitivity analyses were performed by several methods. Galbraith’s plot and leave-one-out analysis were used to display the sources of heterogeneity. HRs were also summarized after excluding studies showing high heterogeneity to test the stability of the pooled results. Subgroup analyses for the relationship of *H. pylori* infection status with overall survival were conducted according to the study-level factors such as study location, retrieval method, and *H. pylori* determination method. Cumulative meta-analysis displayed by a forest plot was used to show the change of the pooled results over time. All analyses were conducted in Stata software (version 12.0, Stata Corp, TX, USA), and a two-tailed *P*-value less than 0.05 was deemed statistically significant.

## Results

### Cohort study

A total of 743 eligible patients were included in our cohort study (sFigure 1). Among these 743 patients, 516 patients (69.4%) were positive for *H. pylori*, while 227 (30.6%) were negative. The *H. pylori*-positive patients had shorter tumor diameters (median 4.0 vs 4.5 cm, *P* = 0.012), and therefore, patients with larger tumor diameters (> 4.5 cm) had a lower proportion of *H. pylori*-positive patients (*P* = 0.042, Table 1). We did not observe any statistically significant association between *H. pylori* infection status and demographics characteristics, such as age (*P* = 0.191) and gender (*P* = 0.088), or other pathological factors, such as depth of invasion (T stage, *P* = 0.828), metastasis of lymph nodes (N stage, *P* = 0.549), TNM stage (*P* = 0.089), or the post-operational chemotherapy rate (*P* = 0.697).

**Table 1:** Comparison of *H. pylori*-positive patients and *H. pylori*-negative patients

Variable	Classification	Positive	Negative	*P*-value
*N* = 743		516 (69.4)	227 (30.6)	
Age (years)		60 (23–84)	61 (35–90)	0.191
Age group	≤65 years	359 (71.1)	146 (28.9)	0.157
	> 65 years	157 (66.0)	81 (34.0)	
Gender	Male	390 (71.2)	158 (28.8)	0.088
	Female	126 (64.6)	69 (35.4)	
Length (cm)		4.0 (0.3–22.0)	4.5 (0.5–13.0)	0.012
Length group	≤4.5 cm	314 (72.4)	120 (27.6)	0.042
	> 4.5 cm	202 (65.4)	107 (34.6)	
Differentiation	Poor	376 (71.2)	152 (28.8)	0.262
	Moderate	138 (65.1)	74 (34.9)	
	High	2 (66.7)	1 (33.3)	
Histological type	Tubular	425 (68.1)	199 (31.9)	0.182
	Signet ring cell	55 (75.3)	18 (24.7)	
	Other	36 (78.3)	10 (21.7)	
T stage	T1	77 (72.6)	29 (27.4)	0.828
	T2	71 (69.6)	31 (30.4)	
	T3	300 (69.3)	133 (30.7)	
	T4	68 (66.7)	34 (33.3)	
N stage	N0	160 (71.1)	65 (28.9)	0.549
	N1	135 (71.8)	53 (28.2)	
	N2	103 (68.7)	47 (31.3)	
	N3	118 (65.6)	62 (34.4)	
TNM	I	98 (69.5)	43 (30.5)	0.089
	II	211 (73.8)	75 (26.2)	
	III	207 (65.5)	109 (34.5)	
Lymphovascular invasion	Negative	156 (70.0)	67 (30.0)	0.844
	Positive	360 (69.2)	160 (30.8)	
Neural invasion	Negative	238 (70.4)	100 (29.6)	0.602
	Positive	278 (68.6)	127 (31.4)	
Post-operational chemotherapy	No	344 (69.9)	148 (30.1)	0.697
	Yes	172 (68.5)	79 (31.5)	

After a median following-up time of 7.93 years (95% CI: 7.74–8.02), 369 patients (49.7%) died, 338 (45.5%) were alive, and 36 patients (4.8%) lost to follow-up. The median survival time was 7.43 years (95% CI: 5.78–9.48) and the five-year survival rate was 55.1% (95% CI: 51.4–58.6%) for these 743 patients with curative tumorectomy.

**Fig. 1:**
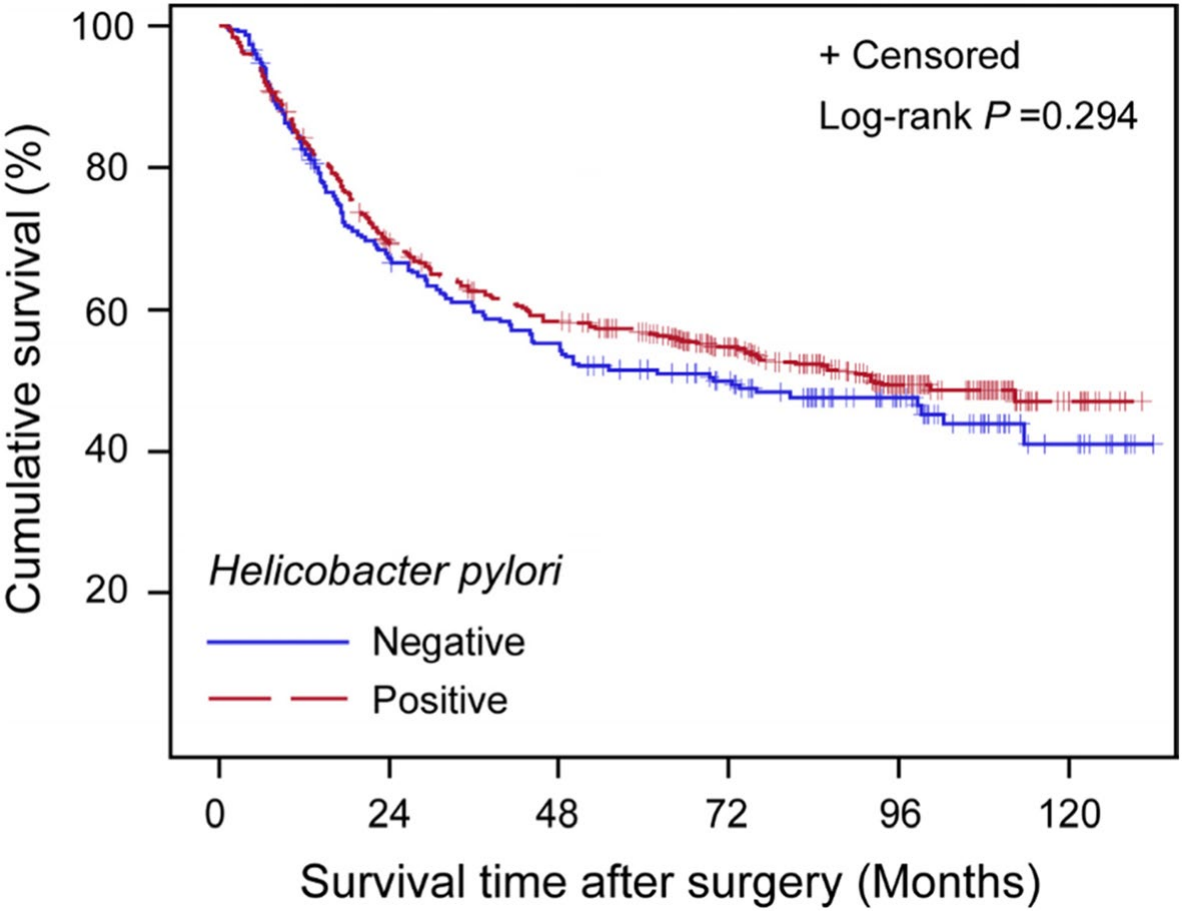
Survival plots for gastric cancer patients of *H. pylori*-positive and -negative

Among 516 patients positive for *H. pylori*, 250 patients (48.4%) died and the median survival time was 7.68 years. Meanwhile, 119 of the 227 *H. pylori*-negative patients (52.4%) died and the median survival time was 5.82 years. The survival curve showed a tendency that the *H. pylori*-positive patients had a slightly lower risk of death, though observed no statistical significance (HR 0.89, 95% CI: 0.72–1.11, *P* = 0.294, Fig. 1). The same nonsignificant trend was observed on the 5-year overall survival: 56.7% (95% CI:52.3–60.9%) for patients of *H. pylori* positive while 51.4% (95% CI: 44.6–57.8%) for patients negative (*P* = 0.185).

**Fig. 2:**
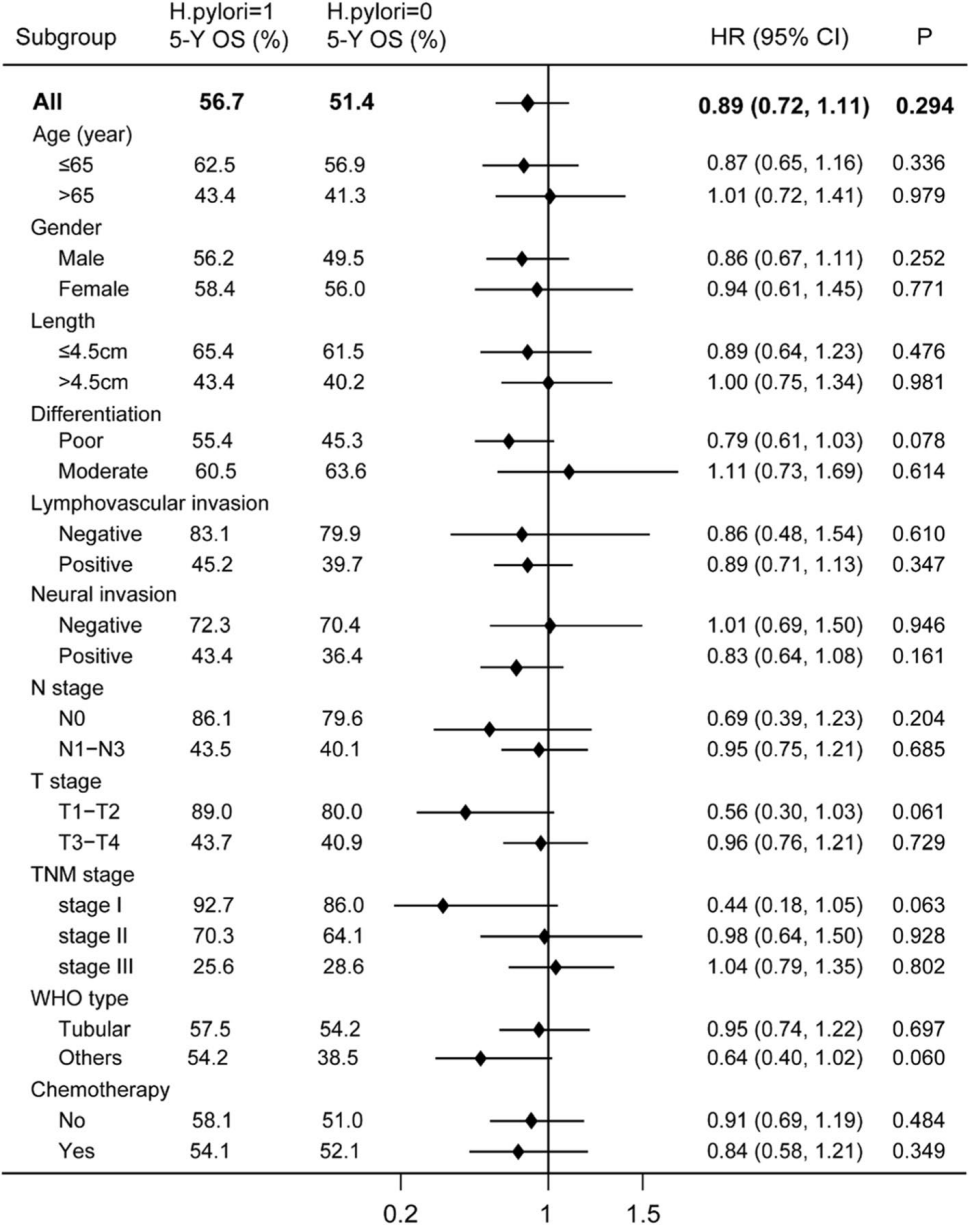
Stratified analysis on the association of *H. pylori* status with gastric cancer overall survival

To clarify the consistency of the relationship between *H. pylori* and GC survival, we performed subgroup analysis stratified by potential prognostic factors (Fig. 2). While there is no significant association was observed in any subgroup. Notably, the point estimation showed a slightly larger trend in patients with less tumor length (≤4.5 cm), and earlier clinical stage (T1-T2 stage, N0 stage, and TNM I stage, Fig. 2).

Further multivariate Cox regression analysis indicated that *H. pylori* status was not significantly associated with the overall survival of GC patients after adjusting for other prognostic factors (HR 0.92, 95%CI: 0.74–1.15, *P* = 0.486). Seven variables were independent predictive factors for OS of gastric cancer: older age (> 65 vs ≤65 years, HR 1.48, 95% CI:1.19–1.84, *P* < 0.001), tumor diameter (> 4.5 vs ≤4.5 cm, HR 1.38, 95%CI: 1.12–1.71, *P* = 0.003), T stage (T3-T4 vs T1-T2, HR 2.46, 95%CI: 1.72–3.51, *P* < 0.001); N stage (N1-N3 vs N0, HR: 2.17, 95%CI: 1.55–3.03, *P* < 0.001), lymphovascular invasion (positive vs negative, HR: 1.82, 95% CI: 1.31–2.55, *P* < 0.001), neural invasion (positive vs negative, HR: 1.35, 95% CI: 1.06–1.71, *P* = 0.016), and postoperational chemotherapy (yes vs no, HR 0.77, 95% CI: 0.61–0.96, *P* = 0.019) (sTable 2).

### Meta-analysis

#### Studies included in the study

**Fig. 3:**
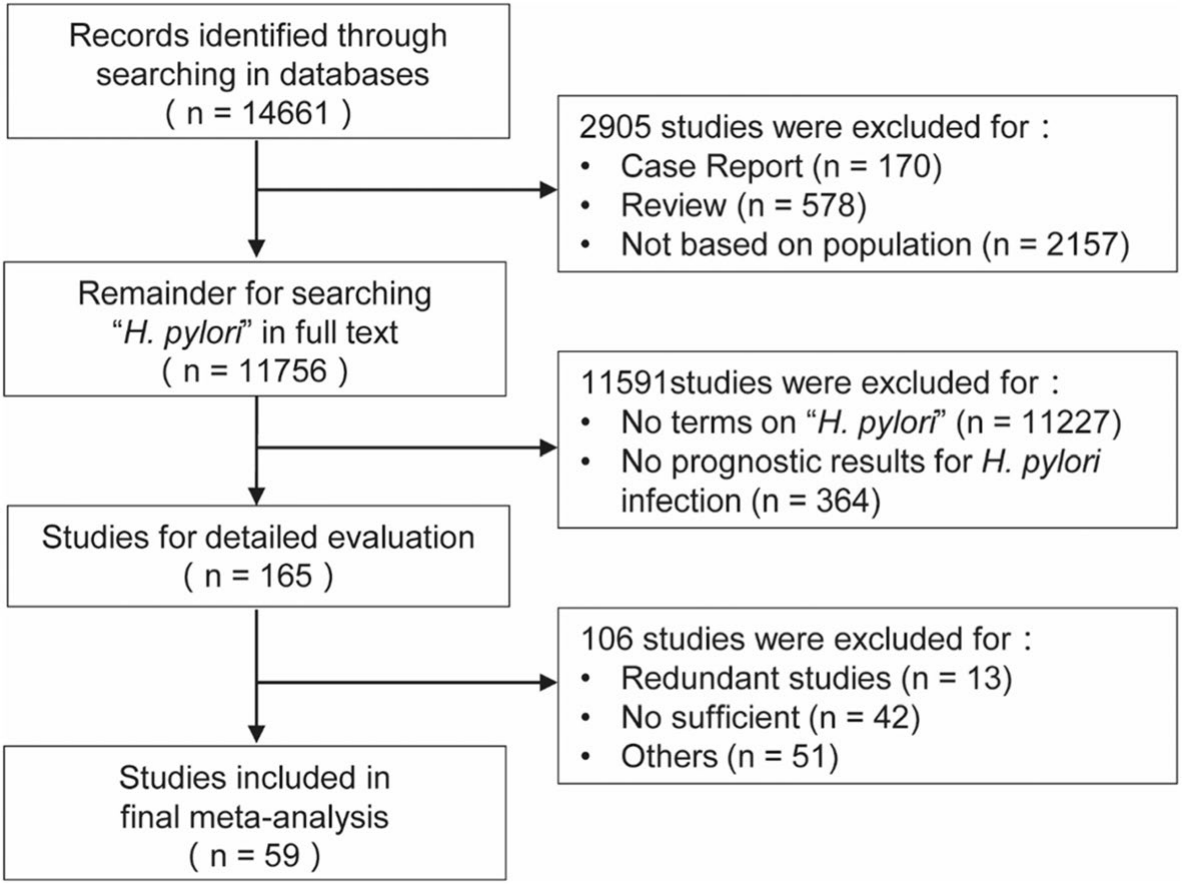
Flow chart of the screening procedure for studies included in the meta-analysis. No terms on “*H. pylori*”: studies without relative terms of “*H. pylori*”; No sufficient: no sufficient data to transform into hazards ratio estimate. Other: studies were excluded by one of the reasons below: (1) some of the subjects were gastric lymphoma; (2) data were from the public databases like TCGA (The Cancer Genome Atlas); (3) the study did not report the relationship of *H. pylori* with a prognosis of gastric cancer

The flow chart of the potential studies appears in Fig. 3. The search of PubMed and Embase databases yielded 14,661 studies. After screening the full texts of these studies, 165 studies reported results on the association of *H. pylori* with GC prognosis. Among them, 59 studies fully met the inclusion and exclusion criteria and were included in the meta-analysis (Table 2).

**Table 2:** Baseline characteristics of the 59 studies included in the meta-analysis

No.	Study	Area	Sample size	Follow-up period (years)	Referring to *H. pylori* ^a^	Mean age (years)	Male (%)	TNM stage	*H. pylori-positive* rate (%)	Samples for *H. pylori* detection	Statistical analysis	NOS score
1	Gong, 2020 [22]	China	200	3.00	Y	32.2	61.0	I-II	78.0	Tissue	U	6
2	Kim, 2020 [23]	Korea	1143	15.00	N	60.1	67.6	I-IV	85.10	Tissue and serum	M	9
3	Tsao, 2020 [24]	America	176	15.00	Y	69	NA	I-III	24.53	Tissue	M	8
4	Xu, 2020 [25]	China	319	10.00	Y	26–84	68.7	I-IV	80.56	Tissue	M	9
5	Zhao, 2020 [26]	China	110	6.67	N	NA	76.4	IV	35.45	NA	U	7
6	Fang, 2019 [7, 27]	Taiwan	356	5.00	Y	NA	69.7	I-III	52.00	Tissue	M	8
7	He, 2019 [28]	China	460	5.00	Y	64	73.5	I-IV	54.6	Serum	U	7
8	Morgan, 2019 [13]	America	249	10.00	Y	65	61.8	I-IV	NA	NA	M	7
9	Shimoda, 2019 [29]	Japan	52	7.67	N	NA	67.3	I-IV	88.00	Tissue	U	8
10	Tahara, 2019 [30, 31]	Japan	214	8.33	N	66	68.2	I-IV	86.4	Tissue	M	8
11	Wang, 2019 [32]	China	956	5.00	N	NA	69.8	I-III	70.6	Serum	U	6
12	Xu, 2019 [33]	China	128	NA	N	59.3	69.5	I-IV	NA	NA	U	6
13	Xue, 2019 [34]	China	102	6.67	N	NA	57.8	I-III	54.9	NA	U	7
14	Liu, 2018 [35]	China	99	5.00	Y	59.5	61.6	NA	70.7	Tissue	U	8
15	Martinson, 2018 [36]	America	52	8.83	Y	59.8	62.1	I-IV	41.3	NA	U	7
16	Nishizuka, 2018 [37]	Japan	491	5.23	Y	NA	67.0	I-III	35.6	Tissue	U	8
17	Park, 2018 [38]	Korea	244	10.00	N	53.8	46.2	NA	49.1	NA	U	**7**
18	Peng, 2018 [39]	China	333	6.00	N	59.4	53.8	I-III	37.2	Medical record	M	7
19	Xu, 2018 [40]	China	280	7.50	N	NA	61.4	I-IV	82.5	Medical record	U	7
20	Jung, 2017 [6]	Korea	314	17.33	Y	55.4	66.9	I-III	40.8	Tissue	M	9
21	Lai, 2017 [8]	China	98	5.00	N	57.0	52.0	I-IV	51.0	Medical record	M	8
22	Lv, 2017 [41]	China	353	7.50	Y	NA	71.1	I-IV	53.5	Serum	U	6
23	Nogueira, 2017 [42]	Portugal	82	3.00	Y	66.6	54.9	I-IV	62.2	Tissue and serum	U	7
24	Tsai, 2017 [43]	Taiwan	1010	12.00	N	63.7	59.6	I-III	19.6	Medical record	U	7
25	Tsai, 2017 [44]	Taiwan	567	10.00	Y	62.2	61.4	I-IV	76.7	Tissue or serum	M	9
26	Xiao, 2017 [45]	China	469	17.00	N	58.9	72.3	I-III	8.96	NA	M	8
27	Chen, 2016 [46]	Taiwan	67	5.00	Y	68.5	70.1	I-IV	65.7	NA	U	6
28	Kim, 2016 [47]	Korea	167	11.30	Y	57.0	69.2	I-IV	50.9	Tissue	M	**9**
29	Liu, 2016 [48]	China	153	7.92	Y	60.0	71.8	I-IV	70.0	Medical record	M	8
30	Liu, 2016 [49]	China	268	8.00	N	NA	NA	I-IV	NA	NA	M	7
31	Postlewait, 2016 [50]	America	559	4.15	Y	64.6	56.0	I-III	18.6	NA	M	8
32	Xu, 2016 [19]	China	1412	5.00	N	64.0	68.6	I-III	78.8	NA	U	7
33	Zhao, 2016 [12]	China	600	10.00	Y	60.0	71.3	I-IV	79.2	Medical record	M	8
34	Zhou, 2016 [51]	China	152	10.00	Y	33.6	34.9	I-IV	51.1	Tissue	U	8
35	Bautista, 2015 [52]	America	802	5.00	Y	66.0	58.0	I-IV	74.1	Medical record	M	8
36	García-González, 2015 [53]	Spain	558	10.33	Y	69.8	68.7	I-IV	68.3	Tissue	M	9
37	Shen, 2015 [54]	Hong Kong	126	NA	Y	61.1	69.3	I-IV	41.3	NA	U	7
38	Wang, 2015 [55]	China	82	2.50	Y	63.3	NA	I-IV	54.0	Tissue	U	7
39	Wei, 2015 [56]	China	166	5.00	Y	62.3	65.1	I-IV	73.5	Medical record	M	8
40	Zhang, 2015 [57]	China	65	3.33	Y	66.7	67.7	I-II	55.4	Serum	M	8
41	Fang, 2014 [58]	China	252	6.50	Y	NA	55.9	NA	84.5	NA	M	7
42	Gong, 2014 [59]	Korea	308	9.35	Y	63.6	70.9	I-IV	84.1	Serum	U	8
43	Jiang, 2014 [18]	China	377	8.58	N	64.0	67.1	I-III	45.9	NA	M	8
44	Posteraro, 2014 [9]	Italy	110	10.00	Y	67.3	54.0	I-IV	78.0	Tissue	U	8
45	Pryczynicz, 2014 [17]	Poland	71	7.00	N	NA	69.0	I-IV	52.1	Tissue	U	8
46	Roberts, 2014 [10]	Jamaica	77	10.92	Y	67.0	45.6	NA	19.5	NA	U	7
47	Suzuki, 2014 [60]	Japan	381	10.00	N	67.1	72.9	I-IV	91.3	NA	M	9
48	Li, 2013 [11]	China	156	5.99	Y	56.0	72.8	I-IV	46.3	Tissue	M	8
49	Wang, 2013 [61]	China	261	4.92	Y	61.0	77.0	I-IV	72.0	Tissue	M	9
50	Choi, 2012 [62]	Korea	61	5.00	Y	57.0	57.4	NA	31.1	Medical record	U	7
51	Hur, 2012 [63]	Korea	174	3.33	Y	NA	37.9	I-IV	63.8	Tissue and serum	U	8
52	Kang, 2012 [64]	Korea	274	15.33	Y	54.0	69.0	I-IV	61.0	Tissue	M	9
53	Syrios, 2012 [65]	Greece	218	5.42	Y	59.0	67.4	I-IV	34.9	Serum	U	6
54	Santos, 2011 [66]	Brazil	68	17.28	Y	59.4	51.5	I-IV	50.0	Tissue	U	7
55	Qiu, 2010 [67]	China	157	6.82	Y	57.2	68.2	I-IV	31.2	Tissue	U	6
56	Marrelli, 2009 [68]	Italy	220	18.33	Y	68.0	58.0	I-IV	89.1	Tissue or serum	M	9
57	Chen, 2006 [69]	Taiwan	79	4.00	Y	63.3	68.4	I-III	38.0	Medical record	M	7
58	Meimarakis, 2006 [70]	Germany	166	12.17	Y	65.0	60.2	I-IV	75.3	Tissue or serum	M	9
59	Lee, 1995 [5]	Taiwan	128	5.00	Y	59.0	57.0	I-IV	64.0	Serum	U	8

^a “^Referring to *H. pylori*” means a paper refers to the terms on *H. pylori* in title, abstract or keyword section

Abbreviations: *NA* not available, *Y* yes, *N* not, *U* univariate method, *M* multivariate method, *NOS* Newcastle-Ottawa Scale

These 59 published studies [5, 6, 8–13, 17–19, 22–30, 32–70], together with our primary study, contained 18,315 GC patients (sFigure 2), with a median sample size of 216 (range: 52–1412). The median percentage of *H. pylori*-positive patients in these studies was 55.4%, ranging from 8.96 to 91.3%. The maximum following-up duration ranged from 2.50 years to 18.33 years. Among these 59 published studies, 18 studies (30.5%) could not be retrieved with the search term *H. pylori*. For the outcome measurements, 56 studies examined overall survival [5, 6, 8–13, 17–19, 22–30, 32–37, 39–46, 48–70], 10 studies examined the disease-free survival (DFS) [9, 24, 27, 34, 37, 39, 48, 57, 61, 63] and 5 studies examined the recurrence-free survival (RFS) [11, 38, 47, 67, 70]. Quality assessment showed that 33 studies had NOS scores > 7 and were classed as a high-quality group, and 26 studies with NOS scores ≤7 were classified as the low-quality group. For the locations of the subjects, 45 studies with 14,164 patients were performed in East Asia, and 14 studies with 3408 patients were carried out in other regions. For the *H. pylori* determination methods, 20 studies used histological-based methods such as hematoxylin-eosin staining, immunohistochemistry, PCR, or urea breath test; 7 studies used the serum-based methods such as ELISA; 6 studies used methods based on more than one kind of specimen; 16 studies did not report the exact method of detection.

#### *H. pylori*-positive status predicted better overall survival

**Fig. 4:**
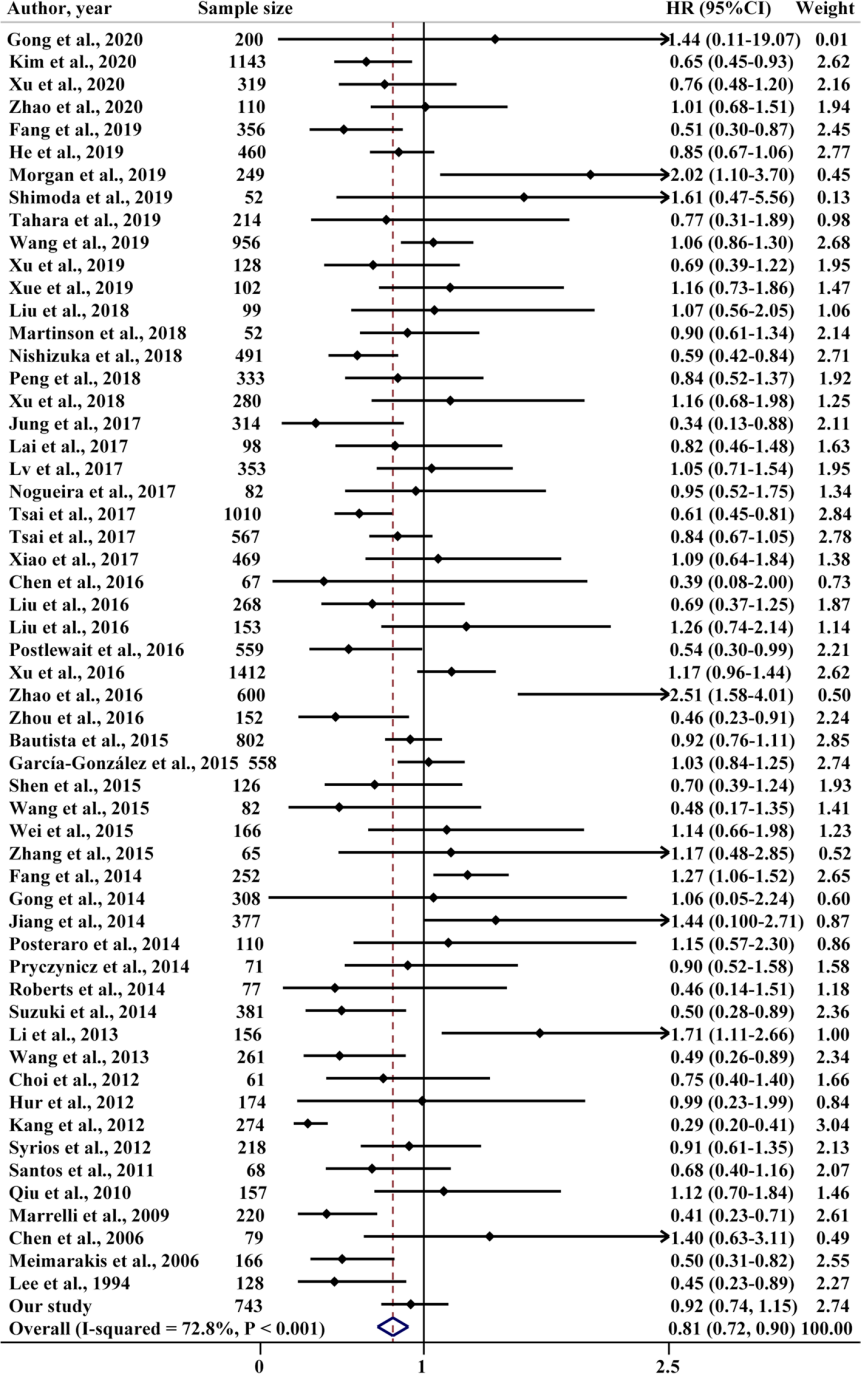
Forest plot on the association of *H. pylori* with OS for gastric cancer patients

**Fig. 5:**
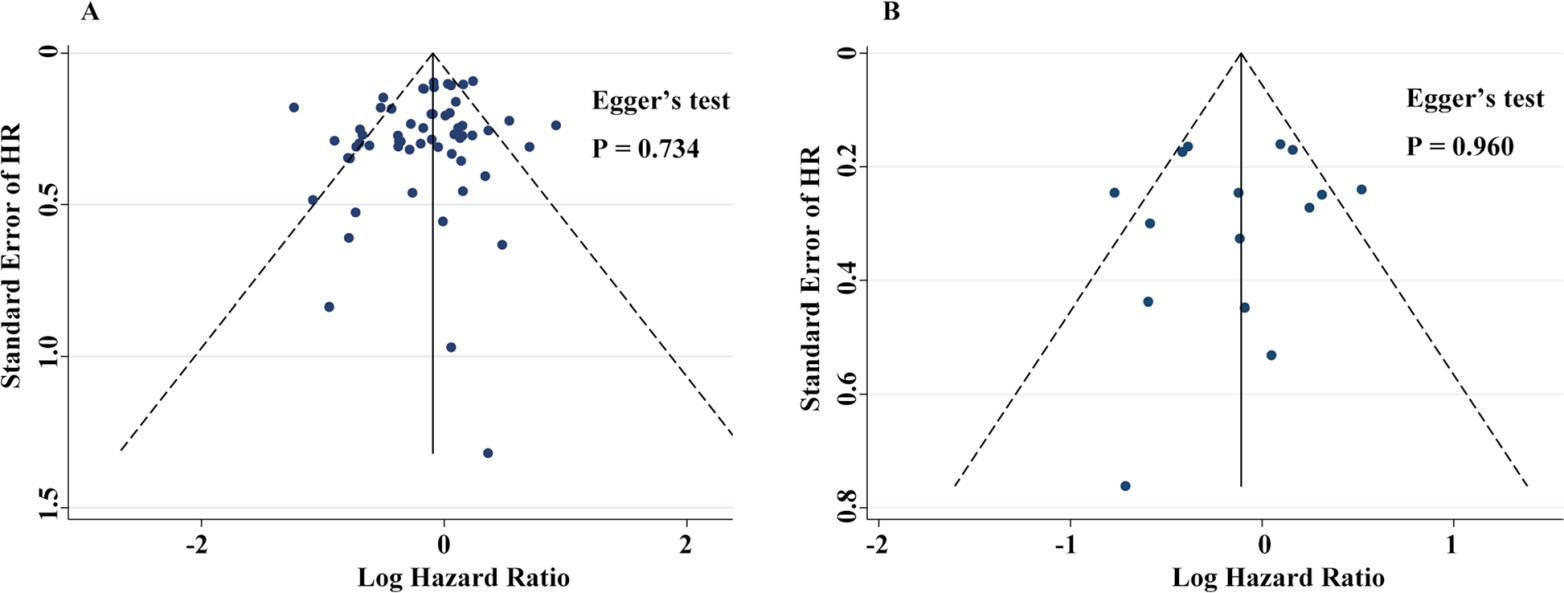
Funnel plot of evaluation of study bias on the association of *H. pylori* status at diagnosis with OS (A) and DFS (B) for gastric cancer patients

A total of 57 studies, including 56 previously published and our primary cohort study, were included in the meta-analysis on the overall survival of GC. These 57 studies included 17,728 gastric cancer patients. The random-effect model was utilized to summarize the results accounting for the heterogeneity among these studies (*I*^*2*^ = 73.8%, *P* < 0.001). The pooled HR was 0.81 (95% CI: 0.72–0.90, *P* < 0.001), indicating that GC patients of *H. pylori*-positive had better OS than patients of negative (Fig. 4). There was no significant bias as shown in funnel plots and Egger’s test (*P* = 0.734, Fig. 5).

Given the significant heterogeneity among studies (*I*^*2*^ = 72.8%; Q = 205.85, *P* < 0.001), we used a graphical method, i.e., Galbraith’s plot, to identify the possible source of the heterogeneity. The Galbraith’s plot suggested that 6 studies [11, 12, 44, 58, 64, 68] were significant sources of heterogeneity (sFigure 3). When these 6 studies were excluded, we observed a significant reduction in heterogeneity (*I*^*2*^ = 43.3%; Q = 88.18, *P* = 0.001), while the pooled effect did not change substantially (HR 0.80, 95%CI: 0.73–0.88, *P* < 0.001) (sFigure 6). The leave-one-out analysis also did not show a substantial change of the pooled effect (sFigure 4), and cumulative meta-analysis also indicated that the summarized effect was temporally stable (sFigure 5).

Further stratified analyses were conducted to examine the stability of the overall estimate and identify the potential study-level influential factors. When stratified by retrieval method, the pooled effect from 17 pieces of research (HR 0.86, 95%CI: 0.73–1.00), which could not be retrieved using the relevant terms related to *H. pylori* and could only be obtained by checking the full texts, was weaker than the effect among studies retrieved by using the terms of *H. pylori* (HR 0.78, 95% CI: 0.66–0.91. Table 3). The results from the subgroup of the 25 low-quality studies (NOS scores≤7), of which the lower scores were mainly caused by not reporting the *H. pylori* determination method and the HRs with 95% CIs, showed that *H. pylori* infection was not significantly associated with the OS of GC (HR 0.90, 95% CI: 0.79–1.01). When stratified by the method of *H. pylori* determination, a similar result was observed in the subgroup that did not describe the exact method of *H. pylori* detection (HR 0.91, 95% CI: 0.77–1.04), and further meta-regression analysis also supported this observation. The results stratified by other factors, such as the geographic location of the subjects (East Asia vs others), the sample size of the study (< 200 vs ≥200), source of HRs with 95% CIs (univariate analysis vs multivariate analysis), *H. pylori*-positive rate (< 70% vs ≥70%), and surgery treatment (all patients having curative resection vs others), showed that the pooled effects were relatively stable across these subgroups, although the absolute point estimation varied (Table 3).

**Table 3:** Subgroup analyses for the association of *H. pylori* status at diagnosis with OS of 56 included studies in the meta-analysis

Subgroups	Number of studies (patients)	Pooled overall survival			Heterogeneity		Meta-regression
		HR	95% CI	Weight (%)	*I*^*2*^	*P*	*P*
Referring to *H. pylori* ^a^							0.521
Without	17 (7404)	0.86	0.73–1.00	30.10	49.8%	0.010	
With	39 (9581)	0.78	0.66–0.91	69.04	76.2%	< 0.001	
Region of the study							0.844
East Asia	43 (13753)	0.82	0.71–0.94	74.63	75.0%	< 0.001	
Other	13 (3232)	0.78	0.62–0.94	25.37	60.6%	0.002	
Sample size							0.519
< 200	26 (2781)	0.79	0.67–0.90	38.00	20.6%	0.173*	
≥ 200	30 (14204)	0.80	0.67–0.93	62.00	82.9%	< 0.001	
Maximum follow-up period							0.300
< 7.0 years	27 (7926)	0.87	0.75–0.98	49.32	56.9	< 0.001	
≥ 7.0 years	27 (8805)	0.75	0.61–0.89	46.68	74.2	< 0.001	
Unknown	2 (254)	0.69	0.39–1.22	2.01	0.0	0.975*	
NOS score							0.160
≤ 7	25 (7182)	0.90	0.79–1.01	44.15	44.3%	0.010	
> 7	31 (9803)	0.73	0.61–0.86	55.85	74.2%	< 0.001	
Statistical analysis							0.921
Univariate	29 (7586)	0.82	0.72–0.92	49.20	36.2%	0.028	
Multivariate	27 (9399)	0.81	0.65–0.96	50.80	82.1%	< 0.001	
*H. pylori*-positive rate							0.487
< 70%	32 (7679)	0.76	0.64–0.88	61.38	70.2%	< 0.001	
≥ 70%	21 (8661)	0.87	0.72–1.02	38.62	70.8%	< 0.001	
Unknown	3 (645)	0.83	0.37–1.29	4.40	47.8%	0.147*	
Examining method of *H. pylori*							0.014*
Histological	18 (3934)	0.70	0.52–0.88	31.18	74.7%	< 0.001	
Serological	7 (4872)	0.88	0.70–1.06	13.26	39.8%	0.126*	
Mix	6 (2352)	0.65	0.47–0.83	13.07	52.2%	0.063*	
Unknown	25 (8211)	0.91	0.77–1.04	42.49	58.1%	< 0.001	
Mean age at diagnosis							0.395
< 65	31 (10210)	0.79	0.66–0.91	58.17	73.4%	< 0.001	
≥ 65	12 (2991)	0.74	0.54–0.94	19.70	64.9%	0.001	
Unknown	12 (3465)	0.93	0.74–1.11	22.12	63.9%	< 0.001	
Proportion of male gender							0.447
< 65%	23 (5841)	0.77	0.65–0.90	42.02	61.0%	< 0.001	
≥ 65%	31 (10794)	0.85	0.71–1.00	54.60	78.8%	< 0.001	
Unknown	2 (350)	0.61	0.26–0.97	3.38	0.0%	0.588*	
TNM stage ^b^							0.588
Earlier stage	14 (6723)	0.81	0.63–0.99	24.95	67.6%	< 0.001	
Other	38 (9773)	0.79	0.68–0.91	68.30	72.1%	< 0.001	
Unknown	4 (489)	0.95	0.56–1.34	6.75	58.9%	0.063*	
Curative resection of all patients							0.949
No	34 (9925)	0.84	0.72–0.97	60.64	77.50%	< 0.001	
Yes	22 (7511)	0.79	0.64–0.94	39.36	67.20%	< 0.001	

^a “^Referring to *H. pylori*” means a paper refers to the terms on *H. pylori* in title, abstract, or keyword section

^b^ “Earlier stage” contains studies only including patients diagnosed at stage I, II, or III; “Others” contains studies included patients diagnosed at I, II, III, or IV

Abbreviations: *NOS* Newcastle-Ottawa Scale

#### Positive *H. pylori* status predicted better disease-free survival

**Fig. 6:**
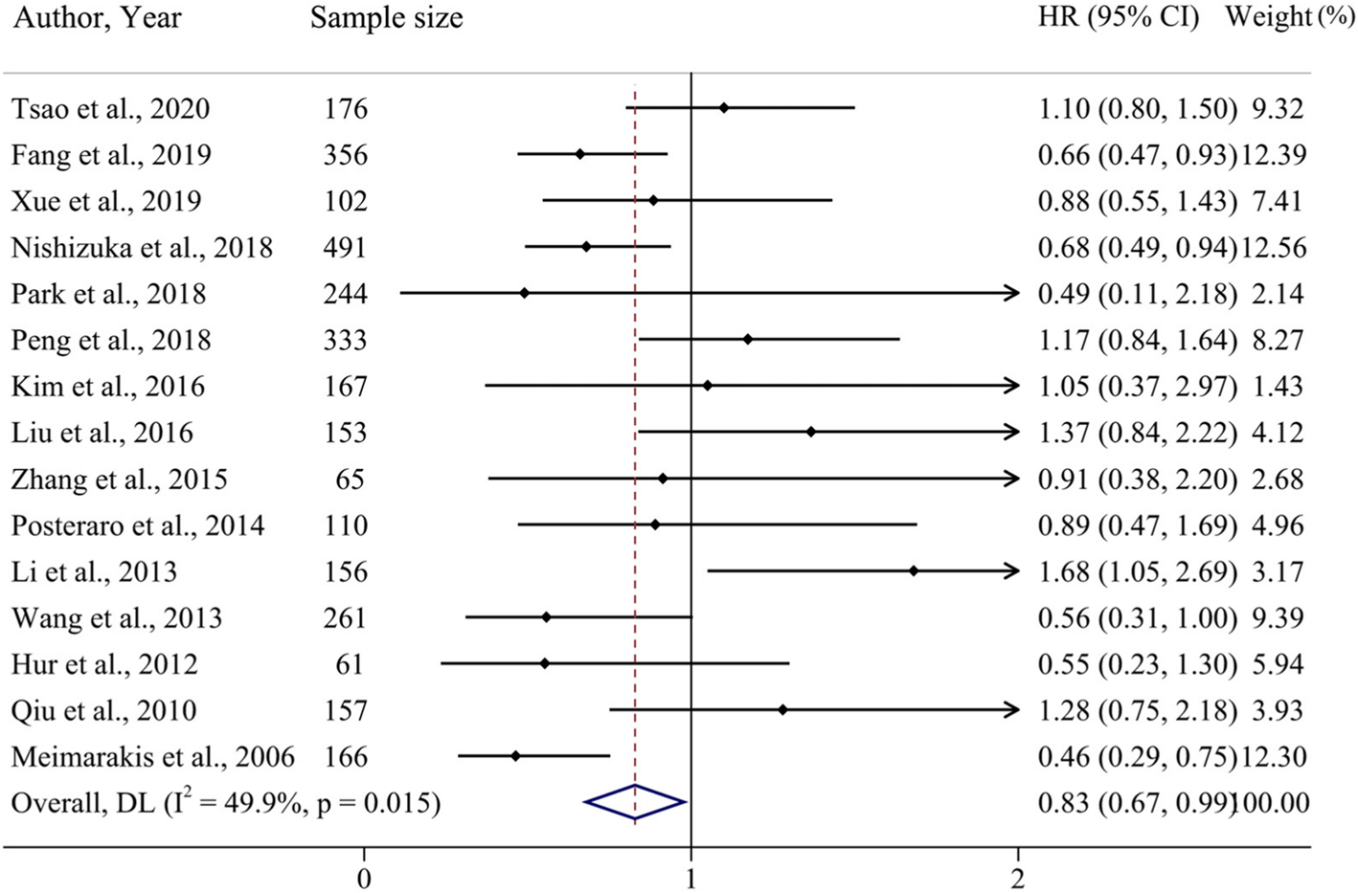
Forest plot for the association of *H. pylori* status at diagnosis with DFS for GC patients

We combined the 10 studies on disease-free survival (DFS, 2221 patients) and 5 studies on recurrence-free survival (RFS, 890 patients) into DFS. The summarized results suggested that the *H. pylori*-positive status might be related to a better short-term outcome of DFS (HR 0.83, 95% CI: 0.67–0.99, Fig. 6), compared to a negative *H. pylori* status. Funnel plots and Egger’s tests revealed that these studies did not indicate significant publication bias (*P* = 0.960, Fig. 5).

## Discussion

*H. pylori* infection can result in chronic gastritis, which can progress to gastric atrophy, intestinal metaplasia, and dysplasia, and subsequently to gastric cancer. Our recent research also observed a significant higher *H. pylori*-positive rate of GC patients than that of nontumor controls. Previous studies on GC suggest a correlation between *H. pylori* status and the prognosis of gastric cancer, but there are no concrete conclusions regarding this association. In this study, by combining the results from our primary cohort study and pooled estimates from a meta-analysis, we reported that positive *H. pylori* status associated with a better prognosis of gastric cancer and that the risk of death was 18% lower among *H. pylori*-positive patients than *H. pylori*-negative ones (HR 0.82, 95% CI:0.72–0.91).

In our primary cohort study, we observed the trend that the survival of *H. pylori*-positive patients was better than that of *H. pylori*-negative patients from the survival plot (Fig. 1). However, the difference did not reach the significant threshold. Though the relatively large number of patients included (*N* = 743), the post hoc analysis of power pointed out that the test efficacy was only 23.7%, implying an inadequate power at the interpretation of the effect size of HR = 0.92 at the current sample size for our study. To increase the test power by obtaining a much larger sample size, we combined the results of the relevant researches to reach a convincing conclusion.

We used an exhaustive strategy to search for potential studies by checking all the full papers on the prognosis of gastric cancer. This allowed us to find 18 studies not referring to *H. pylori* in the abstract that were not included in the previous meta-analyses [15, 16]. Three of these papers examined DFS [34, 38, 39] and 17 examined OS [8, 17–19, 23, 26, 29, 30, 32–34, 39, 40, 44, 45, 49, 60]. Although subgroup analysis showed that these papers did not change the conclusion substantially, the association magnitude was relatively lower (Table 3). Therefore, searching strategy is critically important to cover all the relevant studies in systematic reviews.

In the pooled analysis, we observed that *H. pylori*-positive patients had longer OS and DFS than *H. pylori*-negative patients. We also observed the protective role of *H. pylori* on OS both in East Asian and non-East Asian patients, which differed from the findings of the recent meta-analyses that included a relatively small number of studies [15, 16]. For the detection method, however, we observed that the subgroup of studies that did not describe the exact method of detection indicated no association between *H. pylori* status and prognosis of GC patients, while subgroups of studies using serum-based or tissue-based methods of detection showed very similar associations (Table 3).

The mechanisms underlying the association between positive *H. pylori* status and better prognosis have yet to be elucidated. Two explanations may partially explain the association. The first is that *H. pylori*-related GC might be different from that of non- *H. pylori*-related GC [71, 72]. The expression profiles of *H. pylori*-positive GC are different from those of *H. pylori*-negative GC at both the mRNA and protein levels. These genes are involved in cancer-related pathways such as the ERK/MAPK, and Wnt/β-catenin signaling pathways, which may result from the epigenetic changes and the chronic inflammation induced by *H. pylori* infection. The second is the immunologic response induced by *H. pylori*. Bacterial virulence factors of *H. pylori*, such as cytotoxin-associated gene A (CagA) and vacuolating cytotoxin A (VacA), not only facilitate colonization of the bacterium in gastric mucosa but also induce innate and adaptive immune responses. Previous studies have revealed that individuals positive for *H. pylori* have a lower risk of some allergic diseases such as asthma [73]. *H. pylori*-induced immune responses mainly involve cell-mediated immunity, in which T helper cell 1 (Th1) was activated [74]. *H. pylori*-positive patients with tumorectomy had higher levels of Th1 cells, and a high level of Th1 is associated with a favourable outcome of GC [75].

Further evidence suggested that the *H. pylori* would be driven out of the gastric mucosa with the progression of *H. pylori*-related gastric atrophy and less production of acid by the atrophic mucosa [43, 76]. Some infected individuals are naturally eradicated, and the *H. pylori* rate decreases [42]. Moreover, *H. pylori* eradication has limited effects in reducing gastric cancer risk for those infected individuals who have already progressed to preneoplastic lesions as the biological change caused by *H. pylori* could not be reversible [31], which suggests that some *H. pylori-*related gastric cancer patients would be detected as *H. pylori*-negative when diagnosis. Our results suggest that patients positive for *H. pylori* are prone to have less tumor length (≤4.5 cm) and early-stage (T1) disease, although without statistical significance for the difference (Table 1). This trend has also been observed in other studies [77]. When conducting an analysis stratified by these factors, the association showed a tendency to be more prominent in less tumor length (≤4.5 cm) and less advanced T1-T2 stage (Fig. 2). Therefore, the magnitude of the effect of *H. pylori* on the prognosis of GC might be underestimated, as the “false negative” patients would confound the result to the direction of no association.

Three limitations should be addressed in our study. First, heterogeneity between included studies existed in the analysis. We used several strategies to account for the heterogeneity such as subgroup analysis and sensitivity analysis, however, we could not exclude the heterogeneity resulting from the differences in patient ethnicity, *H. pylori* detection method, and other possible non-identified factors. Second, we did not measure DFS, in our primary cohort study. We summarized the relevant studies in the meta-analysis and found that *H. pylori* exerted a protective role on DFS. Third, although a more exhaustive search strategy had been developed, we found out another 11 potential studies that only drew a conclusion or reported a *P*-value related to whether *H. pylori* infection could affect the prognosis of the GC population. These results were ineligible and could not be summarized in this meta-analysis.

## Conclusions

In conclusion, the combined results from our primary cohort study and meta-analysis show that *H. pylori*-positive patients have a better prognosis compared with *H. pylori-*negative patients. More aggressive treatment and stringent surveillance strategies will be necessary for patients negative for *H. pylori* patients at cancer diagnosis.

## Supplementary Information

**Additional file 1: sTable 1.** The modified Newcastle-Ottawa quality assessment scale used for assessing the quality of the studies included in meta-analysis. **sTable 2.** Multivariable analysis of variables associated with the OS of patients in our cohort study. **sFigure 1.** Flow chart for patients screening in our cohort study. **sFigure 2.** Number of studies included in meta-analysis section. n: Number of gastric cancer patients. OS: overall survival. DFS: disease-free survival. RFS: relapse-free survival. **sFigure 3.** Galbraith’s plot for the association of *H. pylori* status at diagnosis with OS for GC patients. **sFigure4.** Leave-one-out analysis for the association of *H. pylori* status at diagnosis with OS for GC patients. **sFigure 5.** Cumulative meta-analysis for the association of *H. pylori* status at diagnosis with DFS for GC patients. **sFigure 6.** Forest plot for the association of *H. pylori* status with OS on GC (6 studies removed version). 6 studies removed: This version of forest plot displays result of meta-analysis when 6 studies were removed to reduce the heterogeneity.

**Additional file 2.**

## Data Availability

The data used in the Cohort Study section can be openly accessed for a non-commercial purpose in supplementary file 2. And the data generated in the section of the meta-analysis are included in our study and referenced articles are listed in the References section (Table 2),

## Abbreviations

GC: Gastric cancer
HR: Hazard ratio
AJCC: The American Joint Committee on Cancer
IgG: Immunoglobulin G
ELISA: Enzyme-linked immunosorbent assay
EIU: Enzyme immunoassay units
SD: Standard deviations
CI: Confidence interval
OS: Overall survival
DFS: Disease-free survival
RFS: Recurrence-free survival
NOS: Newcastle-Ottawa Scale
NA: Not available
Y: Yes
N: Not
U: Univariate method
M: Multivariate method
CagA: Cytotoxin-associated gene A
VacA: Vacuolating cytotoxin A
Th1: T helper cell 1

## References

[1] Bray F (2018). Global cancer statistics 2018: GLOBOCAN estimates of incidence and mortality worldwide for 36 cancers in 185 countries. CA Cancer J Clin.

[2] IARC (1994). Schistosomes, liver flukes and Helicobacter pylori. IARC Working Group on the Evaluation of Carcinogenic Risks to Humans. Lyon, 7-14 June 1994. IARC Monogr Eval Carcinog Risks Hum.

[3] Amieva M, Peek RM (2016). Pathobiology of Helicobacter pylori-induced gastric Cancer. Gastroenterology.

[4] Ford AC, Yuan Y, Moayyedi P. Helicobacter pylori eradication therapy to prevent gastric cancer: systematic review and meta-analysis. Gut. 2020;69(12):2113-21. 10.1136/gutjnl-2020-320839 32205420

[5] Lee WJ (1995). Comparison between resectable gastric adenocarcinomas seropositive and seronegative for Helicobacter pylori. Br J Surg.

[6] Jung DH (2017). Postoperative Helicobacter pylori infection as a prognostic factor for gastric Cancer patients after curative resection. Gut Liver.

[7] Fang WL (2019). Comparison of the Clinicopathological characteristics and genetic alterations between patients with gastric Cancer with or without Helicobacter pylori infection. Oncologist.

[8] Lai Y (2017). Decreased expression of the long non-coding RNA MLLT4 antisense RNA 1 is a potential biomarker and an indicator of a poor prognosis for gastric cancer. Oncol Lett.

[9] Posteraro B (2014). Prognostic factors and outcomes in Italian patients undergoing curative gastric cancer surgery. Eur J Surg Oncol.

[10] Roberts PO (2014). Pathological factors affecting gastric adenocarcinoma survival in a Caribbean population from 2000-2010. World J Gastrointest Surg.

[11] Li G (2013). Gastric cancer patients with Helicobacter pylori infection have a poor prognosis. J Surg Oncol.

[12] Zhao W (2016). Trop2 is overexpressed in gastric cancer and predicts poor prognosis. Oncotarget.

[13] Morgan R (2019). Presentation and survival of gastric Cancer patients at an urban academic safety-net hospital. J Gastrointest Surg.

[14] Zheng F (2017). Is it a protective factor of Helicobacter pylori infection in overall survival of all gastric Cancer? Evidence from Meta-analysis. J Environ Pathol Toxicol Oncol.

[15] Li G (2019). The prognostic role of Helicobacter pylori in gastric cancer patients: a meta-analysis. Clin Res Hepatol Gastroenterol.

[16] Fang X (2017). Positive Helicobacter pylori status is associated with better overall survival for gastric cancer patients: evidence from case-cohort studies. Oncotarget.

[17] Pryczynicz A (2014). PRL-3 and E-cadherin show mutual interactions and participate in lymph node metastasis formation in gastric cancer. Tumour Biol.

[18] Jiang N (2014). The role of preoperative neutrophil-lymphocyte and platelet-lymphocyte ratio in patients after radical resection for gastric cancer. Biomarkers.

[19] Xu YQ (2016). Prognostic value of ABO blood group in patients with gastric cancer. J Surg Res.

[20] Edge SB, Compton CC (2010). The American joint committee on Cancer: the 7th edition of the AJCC cancer staging manual and the future of TNM. Ann Surg Oncol.

[21] Tierney JF (2007). Practical methods for incorporating summary time-to-event data into meta-analysis. Trials.

[22] Gong X, Zhang H. Diagnostic and prognostic values of anti-helicobacter pylori antibody combined with serum CA724, CA19-9, and CEA for young patients with early gastric cancer. J Clin Lab Anal. 2020;34(7):e23268. 10.1002/jcla.23268 PMC7370745 32118318

[23] Kim HJ (2020). The influence of family history on stage and survival of gastric Cancer according to the TGFB1 C-509T polymorphism in Korea. Gut and liver.

[24] Tsao MW (2020). The impact of race and socioeconomic status on the presentation, management and outcomes for gastric cancer patients: analysis from a metropolitan area in the Southeast United States. J Surg Oncol.

[25] Xu L (2020). Polyamine synthesis enzyme AMD1 is closely associated with tumorigenesis and prognosis of human gastric cancers. Carcinogenesis.

[26] Zhao G (2020). Prognostic significance of the neutrophil-to-lymphocyte and platelet-to-lymphocyte ratio in patients with metastatic gastric cancer. Medicine.

[27] Fang W-L (2019). Comparison of the Clinicopathological characteristics and genetic alterations between patients with gastric Cancer with or without infection. Oncologist.

[28] He B (2019). Polymorphisms of IL-23R predict survival of gastric cancer patients in a Chinese population. Cytokine.

[29] Shimoda T (2019). Expression of protein disulfide isomerase A3 and its clinicopathological association in gastric cancer. Oncol Rep.

[30] Tahara T (2019). Molecular subtyping of gastric cancer combining genetic and epigenetic anomalies provides distinct clinicopathological features and prognostic impacts. Hum Mutat.

[31] Tahara S (2019). DNA methylation accumulation in gastric mucosa adjacent to cancer after Helicobacter pylori eradication. Int J Cancer.

[32] Wang X-Q (2019). Association of rs2094258 polymorphism with gastric cancer prognosis. World J Gastroenterol.

[33] Xu Z (2019). Comprehensive profiling of JMJD3 in gastric cancer and its influence on patient survival. Sci Rep.

[34] Xue F (2019). 4.1B suppresses cancer cell proliferation by binding to EGFR P13 region of intracellular juxtamembrane segment. Cell Commun Signal.

[35] Liu LP (2018). Helicobacter pylori promotes invasion and metastasis of gastric cancer by enhancing heparanase expression. World J Gastroenterol.

[36] Martinson HA (2018). Gastric cancer in Alaska native people: a cancer health disparity. World J Gastroenterol.

[37] Nishizuka SS (2018). Helicobacter pylori infection is associated with favorable outcome in advanced gastric cancer patients treated with S-1 adjuvant chemotherapy. J Surg Oncol.

[38] Park JC (2018). Long-term outcomes of endoscopic submucosal dissection in comparison to surgery in undifferentiated-type intramucosal gastric cancer using propensity score analysis. Surg Endosc.

[39] Peng W (2018). The correlation of circulating pro-angiogenic miRNAs' expressions with disease risk, clinicopathological features, and survival profiles in gastric cancer. Cancer Med.

[40] Xu B (2018). Significance and prognostic role of human epidermal growth factor receptor 2 and RAB1A expression in gastric cancer. Oncol Lett.

[41] Lv Z (2017). Long non-coding RNA polymorphisms in 6p21.1 are associated with atrophic gastritis risk and gastric cancer prognosis. Oncotarget.

[42] Nogueira C (2017). Prevalence and characteristics of Epstein-Barr virus-associated gastric carcinomas in Portugal. Infect Agent Cancer.

[43] Tsai KF (2017). Distinct Clinicopathological features and prognosis of Helicobacter pylori negative gastric Cancer. PLoS One.

[44] Tsai CY (2017). Comprehensive profiling of virus microRNAs of Epstein-Barr virus-associated gastric carcinoma: highlighting the interactions of ebv-Bart9 and host tumor cells. J Gastroenterol Hepatol.

[45] Xiao J (2017). Prognostic significance of pretreatment serum carcinoembryonic antigen levels in gastric cancer with pathological lymph node-negative: a large sample single-center retrospective study. World J Gastroenterol.

[46] Chen WM (2016). Expression of Helios in gastric tumor cells predicts better survival in gastric cancer patients. J Cancer Res Clin Oncol.

[47] Kim YI (2016). Effect of Helicobacter pylori eradication on long-term survival after distal Gastrectomy for gastric Cancer. Cancer Res Treat.

[48] Liu Y (2016). High PARP-1 expression is associated with tumor invasion and poor prognosis in gastric cancer. Oncol Lett.

[49] Liu Y (2016). Dapper homolog 1 alpha suppresses metastasis ability of gastric cancer through inhibiting planar cell polarity pathway. Oncotarget.

[50] Postlewait LM (2016). Preoperative Helicobacter pylori infection is associated with increased survival after resection of gastric adenocarcinoma. Ann Surg Oncol.

[51] Zhou F (2016). Gastric carcinomas in young (younger than 40 years) Chinese patients: Clinicopathology, family history, and Postresection survival. Medicine (Baltimore).

[52] Bautista MC (2015). Significant racial disparities exist in Noncardia gastric Cancer outcomes among Kaiser Permanente's patient population. Dig Dis Sci.

[53] Garcia-Gonzalez MA (2015). Association of PSCA rs2294008 gene variants with poor prognosis and increased susceptibility to gastric cancer and decreased risk of duodenal ulcer disease. Int J Cancer.

[54] Shen J (2015). Epigenetic silencing of miR-490-3p reactivates the chromatin remodeler SMARCD1 to promote Helicobacter pylori-induced gastric carcinogenesis. Cancer Res.

[55] Wang G (2015). The diagnosis value of promoter methylation of UCHL1 in the serum for progression of gastric cancer. Biomed Res Int.

[56] Wei B (2015). Association between the expression of T-cadherin and vascular endothelial growth factor and the prognosis of patients with gastric cancer. Mol Med Rep.

[57] Zhang C (2015). Expression of secreted phospholipase A2-group IIA correlates with prognosis of gastric adenocarcinoma. Oncol Lett.

[58] Fang N (2014). Clinicopathological characteristics and prognosis of gastric cancer with malignant ascites. Tumour Biol.

[59] Gong EJ (2014). Risk factors and clinical outcomes of gastric cancer identified by screening endoscopy: a case-control study. J Gastroenterol Hepatol.

[60] Suzuki A (2014). Prevalence of synchronous colorectal neoplasms in surgically treated gastric cancer patients and significance of screening colonoscopy. Dig Endosc.

[61] Wang F (2013). Helicobacter pylori infection predicts favorable outcome in patients with gastric cancer. Curr Oncol.

[62] Choi IK (2012). The relationship between Helicobacter pylori infection and the effects of chemotherapy in patients with advanced or metastatic gastric cancer. Cancer Chemother Pharmacol.

[63] Hur H (2012). The effects of Helicobacter pylori on the prognosis of patients with curatively resected gastric cancers in a population with high infection rate. J Korean Surg Soc.

[64] Kang SY (2012). Helicobacter pylori infection as an independent prognostic factor for locally advanced gastric cancer patients treated with adjuvant chemotherapy after curative resection. Int J Cancer.

[65] Syrios J (2012). Survival in patients with stage IV noncardia gastric cancer - the influence of DNA ploidy and Helicobacter pylori infection. BMC Cancer.

[66] Santos RS (2011). Helicobacter pylori has no influence on distal gastric cancer survival. Arq Gastroenterol.

[67] Qiu HB (2010). Relationship between H.Pylori infection and clinicopathological features and prognosis of gastric cancer. BMC Cancer.

[68] Marrelli D (2009). Negative Helicobacter pylori status is associated with poor prognosis in patients with gastric cancer. Cancer.

[69] Chen CN (2006). Expression of inducible nitric oxide synthase and cyclooxygenase-2 in angiogenesis and clinical outcome of human gastric cancer. J Surg Oncol.

[70] Meimarakis G (2006). Helicobacter pylori as a prognostic indicator after curative resection of gastric carcinoma: a prospective study. Lancet Oncol.

[71] Hu Y (2018). Analysis of key genes and signaling pathways involved in Helicobacter pylori-associated gastric cancer based on the Cancer genome atlas database and RNA sequencing data. Helicobacter.

[72] Lian G (2014). Protein profiling of Helicobacter pylori-associated gastric cancer. Am J Pathol.

[73] Gravina AG (2018). Helicobacter pylori and extragastric diseases: a review. World J Gastroenterol.

[74] Bagheri N (2016). Role of regulatory T-cells in different clinical expressions of Helicobacter pylori infection. Arch Med Res.

[75] Jafarzadeh A (2018). T cell subsets play an important role in the determination of the clinical outcome of Helicobacter pylori infection. Microb Pathog.

[76] Kang HY (2006). Progression of atrophic gastritis and intestinal metaplasia drives Helicobacter pylori out of the gastric mucosa. Dig Dis Sci.

[77] Kolb JM (2017). Effect of Helicobacter pylori infection on outcomes in resected gastric and gastroesophageal junction cancer. J Gastrointest Oncol.

