# Supplementary material for: Positive H. pylori status predicts better prognosis of non-cardiac gastric cancer patients: results from cohort study and meta-analysis
Source: BMC Cancer. 2022 Feb 8;22:155. doi: 10.1186/s12885-022-09222-y (PMC8822753; doi:10.1186/s12885-022-09222-y)
Supplement: Supplementary file 1 — Additional file 1: sTable 1. The modified Newcastle-Ottawa quality assessment scale used for assessing the quality of the studies included in meta-analysis. sTable 2. Multivariable analysis of variables associated with the OS of patients in our cohort study. sFigure 1. Flow chart for patients screening in our cohort study. sFigure 2. Number of studies included in meta-analysis section. n: Number of gastric cancer patients. OS: overall survival. DFS: disease-free survival. RFS: relapse-free survival. sFigure 3. Galbraith’s plot for the association of H. pylori status at diagnosis with OS for GC patients. sFigure4. Leave-one-out analysis for the association of H. pylori status at diagnosis with OS for GC patients. sFigure 5. Cumulative meta-analysis for the association of H. pylori status at diagnosis with DFS for GC patients. sFigure 6. Forest plot for the association of H. pylori status with OS on GC (6 studies removed version). 6 studies removed: This version of forest plot displays result of meta-analysis when 6 studies were removed to reduce the heterogeneity. [file 12885_2022_9222_MOESM1_ESM.docx]

Positive *H. pylori* status predicts better prognosis of non-cardiac gastric cancer patients: results from cohort study and meta-analysis

**Zhifang Jia^1,a^, Min Zheng^1,a^, Jing Jiang^1,3^, Donghui Cao^1^, Yanhua Wu^1^, Yuzheng Zhang^1,3^, Yingli Fu^1^, Xueyuan Cao^2,*^**

^1^ Division of Clinical Research, the First Hospital of Jilin University, Changchun, China

^2^ Department of Gastrointestinal Surgery, the First Hospital of Jilin University, Changchun, China

^3^ Department of Epidemiology and Biostatistics, School of Public Health, Jilin University, Changchun, China

**^a^ Contributed equally to this work.**

*Corresponding author. Department of Gastrointestinal Surgery, the First Hospital of Jilin University, Changchun, China.

Corresponding author: Xueyuan Cao

Email address: jd3d2ub@jlu.edu.cn

**Supplementary materials:**

**The detailed retrieving strategies in PubMed and Embase**

PubMed:

((((("Stomach Neoplasms"[MeSH Terms] OR "gastric cancer"[Title/Abstract]) OR "gastric neoplasms"[Title/Abstract]) OR "stomach cancer"[Title/Abstract]) OR "stomach neoplasm"[Title/Abstract]) OR "cancer of the stomach"[Title/Abstract]) AND ("survival"[Title/Abstract] OR "prognosis"[Title/Abstract])) NOT ((((((("review"[Publication Type] OR "clinical conference"[Publication Type]) OR "comment"[Publication Type]) OR "lecture"[Publication Type]) OR "review"[Title]) OR "overview"[Title]) OR "meta-analysis"[Title]) OR "case report"[Title]) AND 1994/1/1:2020/4/10[Date - Publication] filter:english

Embase:

('stomach cancer'/exp OR 'stomach adenoma'/exp OR 'gastric cancer':ti,ab,kw OR 'gastric neoplasm':ti,ab,kw OR 'stomach neoplasm':ti,ab,kw) AND 'survival':ti,ab,kw NOT ('meta analysis':ti,ab,kw OR [meta analysis]/lim OR 'systematic review':ti,ab,kw OR [systematic review]/lim OR 'lecture':ti OR 'case report':ti OR [erratum]/lim OR [letter]/lim OR [note]/lim OR [review]/lim OR [short survey]/lim) AND [embase]/lim AND [1994-2020]/py AND [english]/lim

sTable 1. The modified Newcastle-Ottawa quality assessment scale used for assessing the quality of the studies included in meta-analysis

| Section | Items | Evidence | Score |
| --- | --- | --- | --- |
| Selection of population | Representativeness of the exposed cohort | All patients were diagnosed as gastric cancer and grouped according to *H. pylori* status | 1 |
|  | Selection of the unexposed cohort | All patients were diagnosed as gastric cancer and grouped according to *H. pylori* status | 1 |
|  | Ascertainment of exposure | By certain methods | 1 |
|  |  | From medical records or not mentioned in this article | 0 |
|  | Outcome of interest not present at start of study | Yes/No | 1/0 |
| Comparability | Control for important factor or additional factor | Adjusted for known prognostic factors | 2 |
|  |  | Univariate analysis only | 1 |
|  |  | Calculated by researchers | 0 |
| Internal validity | Outcome assessment | Defined clearly and detected objectively | 1 |
|  | Follow-up long enough for outcomes to occur | Median length of follow-up > 36m | 1 |
|  |  | The outcome of interest was detected among half of subjects | 1 |
|  |  | Length of follow-up was unavailable or not long enough to detect the outcome | 0 |
|  | Adequacy of follow-up of cohorts | More than 80% of target subjects were included in prognostic studies | 1 |
|  |  | 20% of subjects failed to follow up | 0 |

sTable 2. Multivariable analysis of variables associated with the overall survival of gastric cancer in our cohort study

| Variables | HR | 95% CI | *P* |
| --- | --- | --- | --- |
| Age (>65 vs ≤65 years) | 1.48 | 1.19-1.84 | 0.0004 |
| Length (>4.5cm vs ≤4.5cm) | 1.38 | 1.12-1.71 | 0.0029 |
| T stage (T3-T4 vs T1-T2) | 2.46 | 1.72-3.51 | <.0001 |
| N stage (N1-N3 vs N0) | 2.17 | 1.55-3.03 | <.0001 |
| Lymphovascular invasion (positive vs negative) | 1.82 | 1.31-2.55 | 0.0004 |
| Perineural invasion (positive vs negative) | 1.35 | 1.06-1.71 | 0.0163 |
| Post-operational chemotherapy (yes vs no) | 0.77 | 0.61-0.96 | 0.0191 |


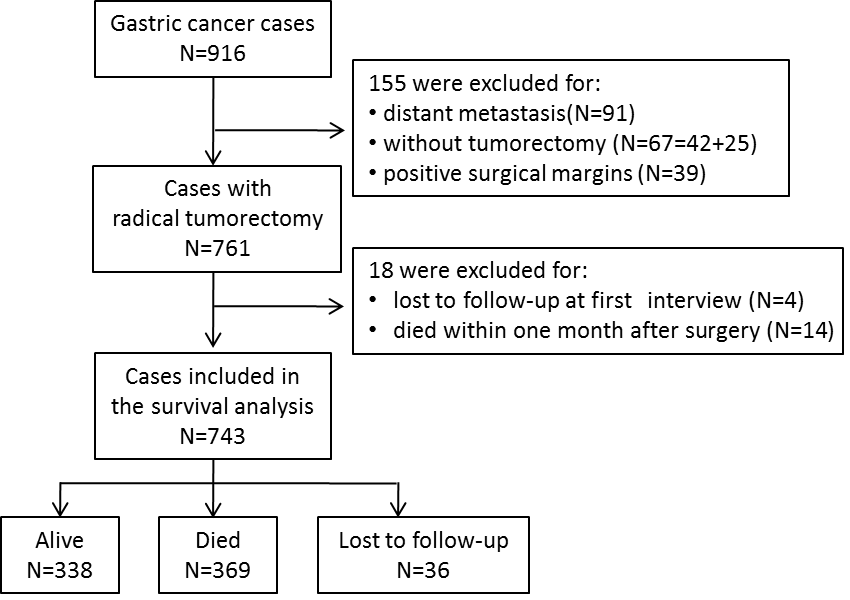


sFigure 1. Flow chart for patient screening in our prospective cohort study


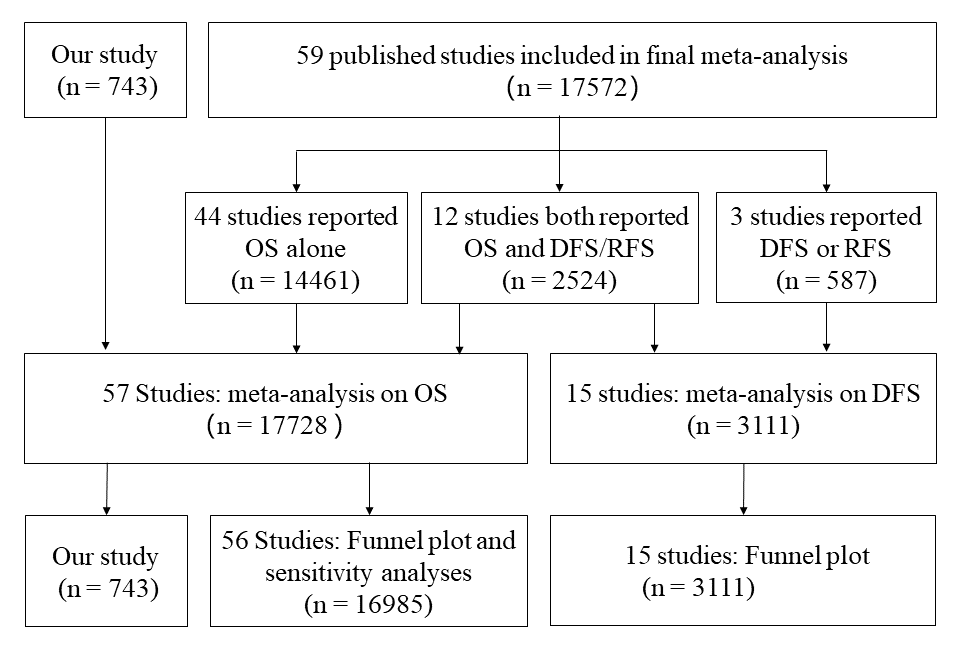


sFigure 2. Number of studies included in meta-analysis section

n: Number of gastric cancer patients. OS: overall survival. DFS: disease-free survival. RFS: relapse-free survival.

s
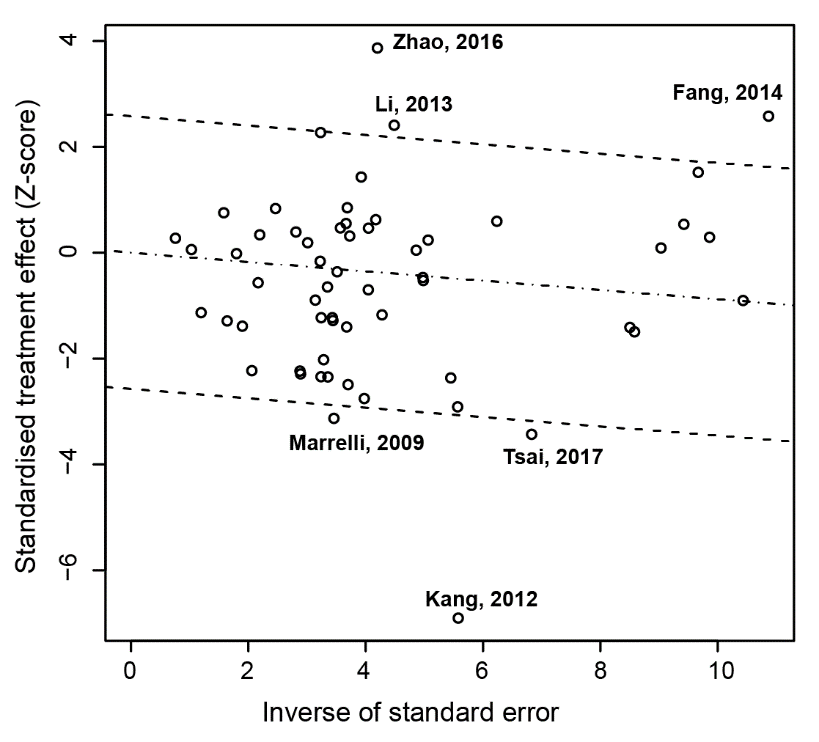
Figure 3. Galbraith’s plot for the association of *H. pylori* status at diagnosis with OS for gastric cancer patients


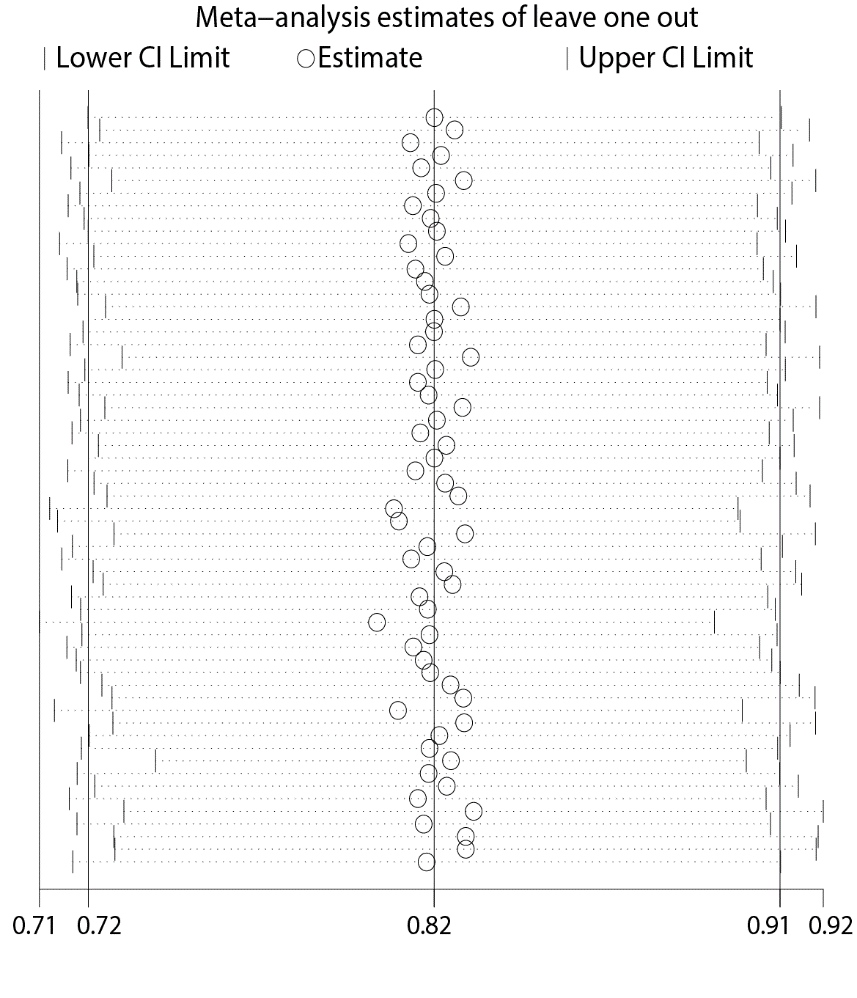
sFigure 4. Leave-one-out analysis for the association of *H. pylori* status at diagnosis with OS for gastric cancer patients


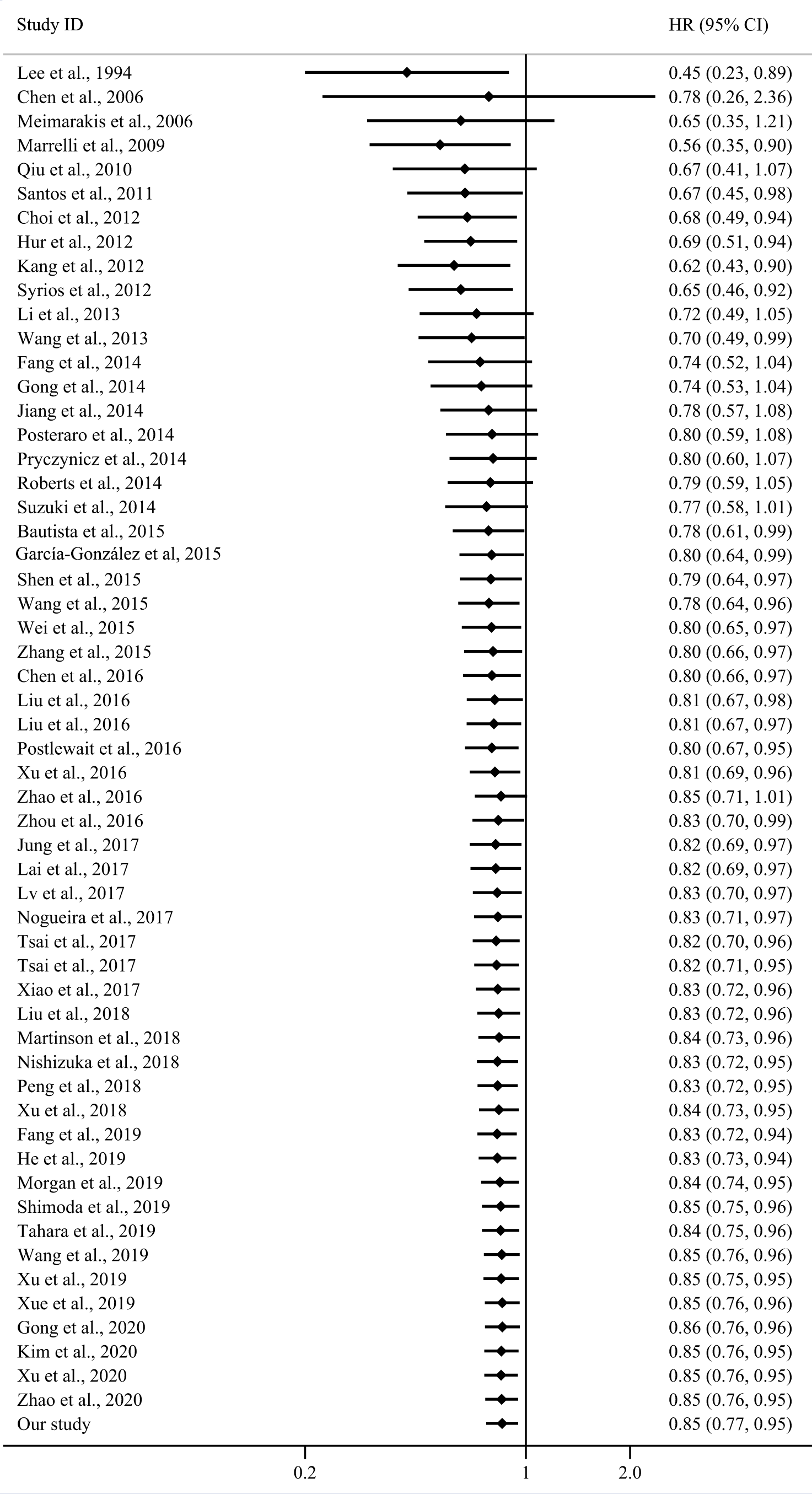


sFigure 5. Cumulative meta-analysis for the association of *H. pylori* status with OS for GC patients


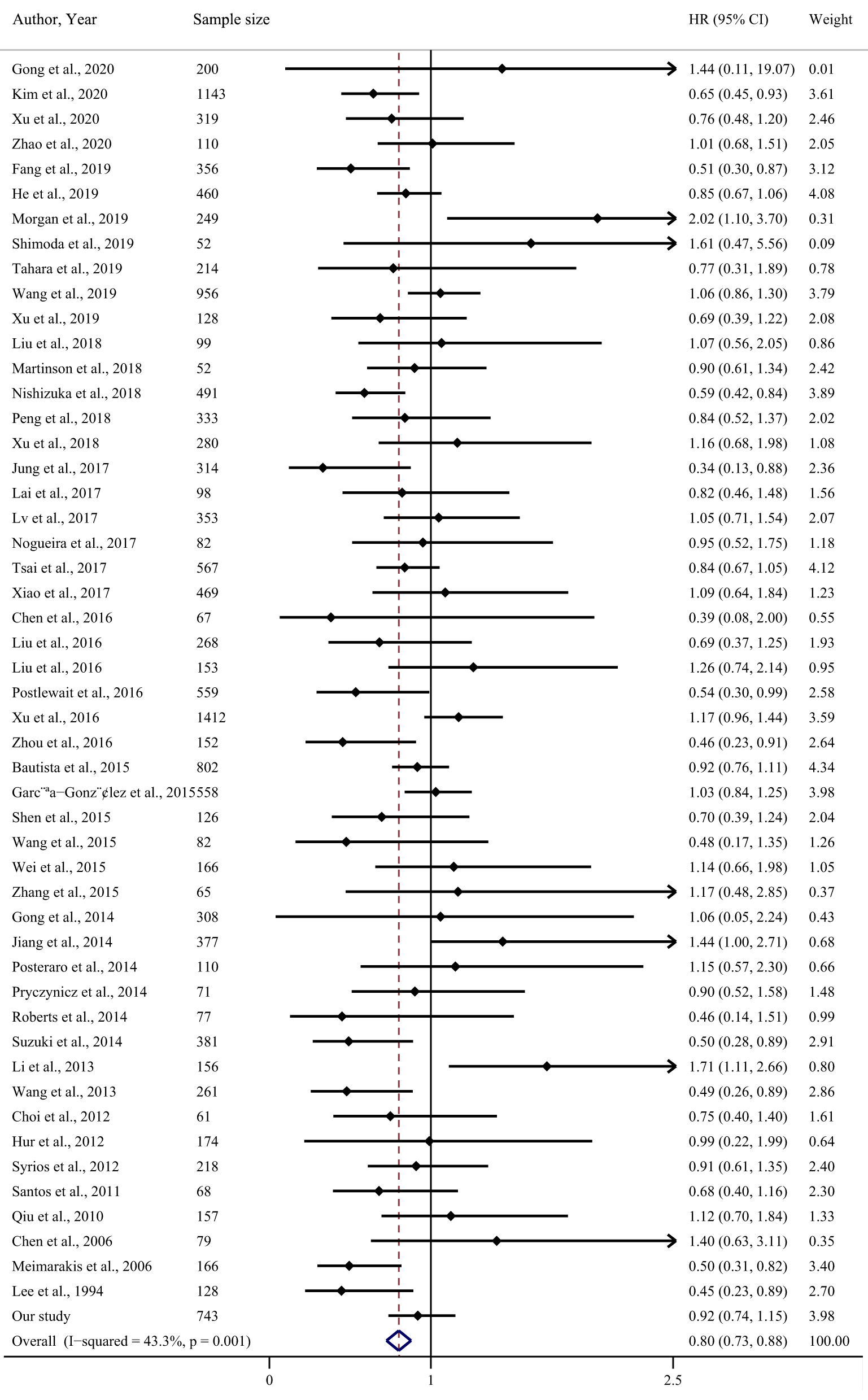


sFigure 6. Forest plot for the association of *H. pylori* status with OS on GC (6 studies removed version)

6 studies removed: This version of the forest plot displays results of meta-analysis when 6 studies were removed to reduce the heterogeneity.
